# The Emancipation from Tuberculosis

**Published:** 1910-02

**Authors:** Theo. Y. Hull

**Affiliations:** San Antonio, Texas


For Texas Medical Journal.

### The Emancipation from Tuberculosis.

BY THEO. Y. HULL, B. S., M. D., SAN ANTONIO, TEXAS.

Is the eradication of tuberculosis possible? Is a clear conception of the real tuberculosis situation attainable by the public mind? Can we enlist the people in an effective movement for its prevention? This is a problem, the solution of which is of the greatest material interest to the student of preventative medicine, to the humanitarian, and to the race.

In an address before the Section on State Medicine of the State Medical Association, I took the position that by the intelligent application of the means at our command it is possible to eradicate tuberculosis in a few decades; and, furthermore, that it is a duty we, as physicians and men, owe to society—both present and future—to do all things within our power to accomplish this end. One of the speakers in discussing the paper held that it is too Utopian to expect such a result.

To my mind the idea that tuberculosis can not be controlled and the natural deduction therefrom that it is useless to attempt it—both of which are inherited ideas—is entirely too pessimistic. This is a form of pessimism or what is sometimes misnamed "conservatism" that has held us back for decades, and with what result? It has acted as a brake on enthusiasm and a check on every advance. We are today at the foot of the incline when we should be half way up or at the top of the hill of prevention. It is time we abandon our position of masterly inactivity, not because it is old but because it is inadequate and accomplishes nothing but self-destruction. We may not be warranted by facts in our present optimism in regard to the outcome in the anti-tuberculosis crusade, but the condition justifies it, for without optimism there can be no enthusiasm, and without enthusiasm there will be little progress. Pessimism is a millstone, while optimism is a life-buoy. Pessimism is closely related to fatalism in that the incentive for the initiation of action is wanting. Optimism looks beyond the present difficulty and believing the thing possible attempts it. In other words, it is hope expressed in action.

Every advance in knowledge extends our vision and broadens our horizon. It makes new undertakings possible. We are doing today things thought impossible a few decades past. One hundred years ago smallpox, typhus fever, cholera and yellow fever were uncontrolled. Inherited ideas classed them as "Visitations of Divine Providence" in chastisement of sin, despite the fact that ignorance and filth—both potent factors in the spread of contagion—were all but universal. These diseases are now practically under control in all civilized portions of the globe, and in two score or more years will be medical curiosities, so complete will be their eradication. Yet when the truth that has made this possible was dawning in the scientific mind and fresh minds conceived new ideas and drew new conclusions from well-known data, the old ignorance held back. The apostle of conservatism held it impossible; therefore, it should not be attempted. The literature of the "Anti-Vaccination Agitation" covers a century, and, when all else failed, the agitators took refuge in metaphysics, where neither logic nor reason could reach them.

We are confronting much the same condition today in our crusade against tuberculosis. This veritable plague has decimated the race for centuries. By long familiarity the populace has come to regard it as inevitable and as a part of the natural order of things. We have become, through lack of knowledge, indifferent to the sorrow and devastation around us. This indifference is rudely awakened, individually, when our own circle is invaded.

It is hardly possible to attain the ideal, for each advance in science changes our conception of the ideal and the attainable, but in attempting it we reach a higher order of things. What was seemingly the ideal a few decades ago is now the ordinary. So it will be in the future. As we approach the fulfillment of one conception, a new conception arises of a higher and more perfect order. It may seem ideal to us, who are accustomed to the fact that every seventh individual dies from tuberculosis, to look forward to a day when less than one in every hundred will share the same fate. Yet that time will come, and in that day they will strive to save the one. It is a part of our nature to strive for it, and we will. Furthermore, the great advances in our knowledge of the nature and mechanism of infection and immunity, warrants us in hoping to attain in the near future a more perfect means of immunizing the healthful against tuberculosis as we do now in smallpox and anthrax.

Tuberculosis is due to the infection of one individual by another. The specific agent—the tubercle bacillus—must pass in some way from one host to another. The work of prevention in this, as in all other infectious and contagious diseases, seeks to do two very important things: to destroy the specific organism or otherwise prevent it from reaching susceptible persons, and to raise the individual power of resistance by sane living or immunization to such a degree that infection does not readily occur. For infection to take place, the pathogenic organism must find lodgment and develop within the living tissues. The normal tissues are inimicable to their growth. When infection takes place, one of two things must have occurred: either the invading organisms have been of sufficient virulence to overcome by their toxins the natural resistance of the normal tissues, or the vitality of the living tissues has been reduced by some cause (predisposition, existing disease, or disturbed nutrition).

The spread of tuberculosis is due to several factors. Chief among these are ignorance, indifference, and avarice. From time immemorial, ignorance has permitted the wanton destruction of human life and still permits it. Formerly the greatest friend of the "White Plague" was ignorance, and it is largely so today. A few decades back there was no knowledge of its contagious nature. It was generally held to be inherited,—a position that some even yet maintain in face of positive evidence. The process of displacing old ideas and of instilling new ones in their stead, is necessarily slow. Only in sudden and overwhelming catastrophe do we find sudden revulsion of feeling and action. Indifference, borne of hopelessness and supposed helplessness, the offspring of ignorance and the lack of a proper valuation of human life, also has had its baneful influence. Year after year and age after age, epidemic came and went strewing its course with human wreckage until the communus vulgus, like dumb driven beasts, simply bowed the head to the blow. This indifference to the loss of human life from supposed incurable diseases is manifest in the inequalities of our laws. If a railroad undertakes to convey one a certain distance and he is injured en route, he may through the courts recover damages; but if the owner or agent of a house, formerly occupied by a case of tuberculosis, leases it to an individual as a dwelling and one or more members of his family contract and die from the disease, he has no recourse whatever. If one knows of an intended murder and does nothing to prevent it, he is accessory before the fact and guilty with the perpetrator; but he as owner or agent of the premises "to let" may have every knowledge of the contagious nature of the malady of the former occupant and makes no effort to either disinfect the premises or notify the would-be tenant of his danger, and he is not guilty of even criminal negligence in the eyes of our lawmakers. Morally, there is just as great a crime in the one instance as the other, but legally there is a vast difference between shooting one's neighbor to death and giving him tuberculosis which insures the same result. Avarice, the third member of this conspiracy against public health—avarice—the brutal intelligence, that taking advantage of ignorance and indifference sacrifices human life for gain—is no negligible factor in the spread of tuberculosis. The crowding of employes, sick and well, in small half-ventilated, half-lighted, and wholly unsanitary apartments, is not altogether uncommon in the so-called age of enlightenment. The housing of the poor and many of the laboring classes, under conditions that invite disease, is likewise not infrequent. The selling of contaminated food and the exposure of food products to contamination is largely unprevented. So common is this practice in many parts of the country that even the most enlightened have no protection. The greed for gain, or call it what you may, that exposes tuberculous meat for sale, that distributes milk from tuberculous cows, and adulterates food, is the same spirit that enters and robs the house at night, burns down a block for the insurance or waylays the belated traveler and slays him for his purse; only on the one hand it is called "business" and on the other "crime."

The practical question is this: can we enlighten the ignorant, arouse the indifferent, and curb the avaricious? It will cost money and effort to do so, but the result will be beyond price. If we can, it is not Utopian to expect to eradicate tuberculosis. For some years a few sporadic and spasmodic attempts have been made to arouse public sentiment and with some results. It remained for the International Tuberculosis Congress, held last autumn in Washington, to crystallize that sentiment and initiate a movement that is now working through all kinds of organizations—State, municipal, labor, commercial, philanthropic, religious, and scientific—in every part of the Union. Such a movement has a vast educational value in creating a healthful public opinion. On August 1, 1908, there were over two hundred anti-tuberculosis societies. There are probably twice that number today. In many States, the boards of health are conducting active campaigns. In others it is being taken up in the public schools, and it is safe to predict that in ten years it will be a part of the regular course of instruction in all the States. The anti-tuberculosis movement is gaining momentum day by day. Its leaders are beginning to realize that the public needs but to be fully enlightened to the enormity of the tuberculosis situation, to arise and demand the enactment and the enforcement of necessary laws.

The situation today is simply this: can we create an enlightened public sentiment sufficiently strong to sustain an energetic movement for the betterment of the home; for the improvement of labor conditions; for the prevention of the sale of contaminated food; for a better system of municipal sanitation; and for the systematic instruction of the young in the cause and prevention of tuberculosis?

I am optimistic enough to believe it possible, and, therefore, feel it my duty to add what strength I can to this great movement for the emancipation of the human race from the thraldom of the "White Plague."

